# Use of a Therapeutic, Socially Assistive Pet Robot (PARO) in Improving Mood and Stimulating Social Interaction and Communication for People With Dementia: Study Protocol for a Randomized Controlled Trial

**DOI:** 10.2196/resprot.4189

**Published:** 2015-05-01

**Authors:** Ruby Yu, Elsie Hui, Jenny Lee, Dawn Poon, Ashley Ng, Kitty Sit, Kenny Ip, Fannie Yeung, Martin Wong, Takanori Shibata, Jean Woo

**Affiliations:** ^1^Department of Medicine and TherapeuticsThe Chinese University of Hong KongHong KongChina (Hong Kong); ^2^Department of Medicine and GeriatricsShatin HospitalHong KongChina (Hong Kong); ^3^Occupational Therapy DepartmentShatin HospitalHong KongChina (Hong Kong); ^4^National Institute of Advanced Industrial Science and TechnologyTokyoJapan; ^5^Tokyo Institute of TechnologyTokyoJapan; ^6^AgeLab, Massachusetts Institute of TechnologyCambridge, MAUnited States

**Keywords:** dementia, elderly, PARO, randomized controlled trial, robot-assisted therapy, socially assistive robots

## Abstract

**Background:**

Socially assistive robotics is a growing area for geriatric research.

**Objective:**

This single-blind, randomized controlled trial (RCT) aims to investigate the use of PARO, a therapeutic, socially assistive pet robot, in improving mood, and stimulating social interaction and communication for people with dementia in the community.

**Methods:**

For the study, 40 community-dwelling older Chinese adults (≥60 years) with mild to moderate dementia will be recruited and randomly assigned to the PARO therapy group or the psychosocial activities control group. Both treatments consist of six, 30-minute weekly sessions, which will be conducted in a geriatric day hospital. Subjects in both groups will be assessed by a trained research assistant at baseline (pre-), during, and post-treatment. Mood (assessed with a simplified face scale), social interaction, and communication (ie, facial expressions and reactions towards each treatment, assessed with an observation table) will be the primary outcome measures. Secondary outcome measures will include assessments on cognitive function (Mini-Mental State Examination) and depressive symptoms (Cornell Scale for Depression in Dementia), as well as caregiver burden (Zarit Burden Inventory). Subjective impression towards each treatment and qualitative comments from the caregivers, facilitator, and therapists will also be obtained.

**Results:**

Recruitment to the pilot study began in 2014 and the last subject is expected to complete their post-treatment assessment in 2015.

**Conclusions:**

This will be the first RCT using PARO to improve mood, and stimulate social interaction and communication in the care of older people with dementia, as well as provide an evidence basis for the use of PARO in dementia care in Hong Kong.

**Trial Registration:**

The Australian New Zealand Clinical Trials Registry (ANZCTR): ACTRN12614000037606; https://www.anzctr.org.au/Trial/Registration/TrialReview.aspx?ACTRN=12614000037606 (Archived by WebCite at http://www.webcitation.org/6Xi7uXdu9).

## Introduction

With a rapidly ageing population, dementia has become an important public health issue worldwide [1]. In Hong Kong, there are over one million people aged 60 and above who had dementia in 2009, and by the year 2039, this is projected to increase to over three million [2]. Non-pharmacological interventions are recommended as the preferred first-line treatment for people with dementia [3,4], with cognitive stimulation therapy being the most studied [5]. The finding from a recent systematic review of cognitive stimulation therapy on optimizing cognitive function of older adults with mild to moderate dementia was comparatively robust and promising. However, the majority of studies have focused on cognitive abilities (eg, memory performance, problem solving, and communication techniques), and the effects on psychological and social aspects remain inconclusive [6,7].

Activities, especially in group settings such as social support groups, may be of psychological and social benefits to people with dementia by reducing depression and improving quality of life [8]. Yet, a great challenge remains with respect to how to stimulate these older people to respond and participate in such activities. Furthermore, owing to a lack of manpower for many day care centres in Hong Kong [9], few provide stimulating social activities for those with dementia. As a result, older adults with dementia spend most of the time sitting around in an environment that is not home, only effectively providing respite care for their family caregivers. Inadequate social engagement can be detrimental, as it magnifies the feelings of loneliness that often accompany the progression of dementia [10]. Other studies have also associated low social engagement with cognitive decline [11] and increased mortality [12].

In recent years, socially assistive robots have been developed for elderly care, particularly companion robots [13,14]. Anecdotal reports, as well as two systematic reviews to date, suggest that robot-assisted therapy is a potentially cost-effective treatment for dementia as it has the potential to improve mood, encourage social interaction and communication, and therefore enhance the well-being of the elderly, and decrease the workload of their caregivers [15,16]. Furthermore, the reported psychosocial effects have been more striking than the results achieved by conventional therapies [17].

**Figure 1:**
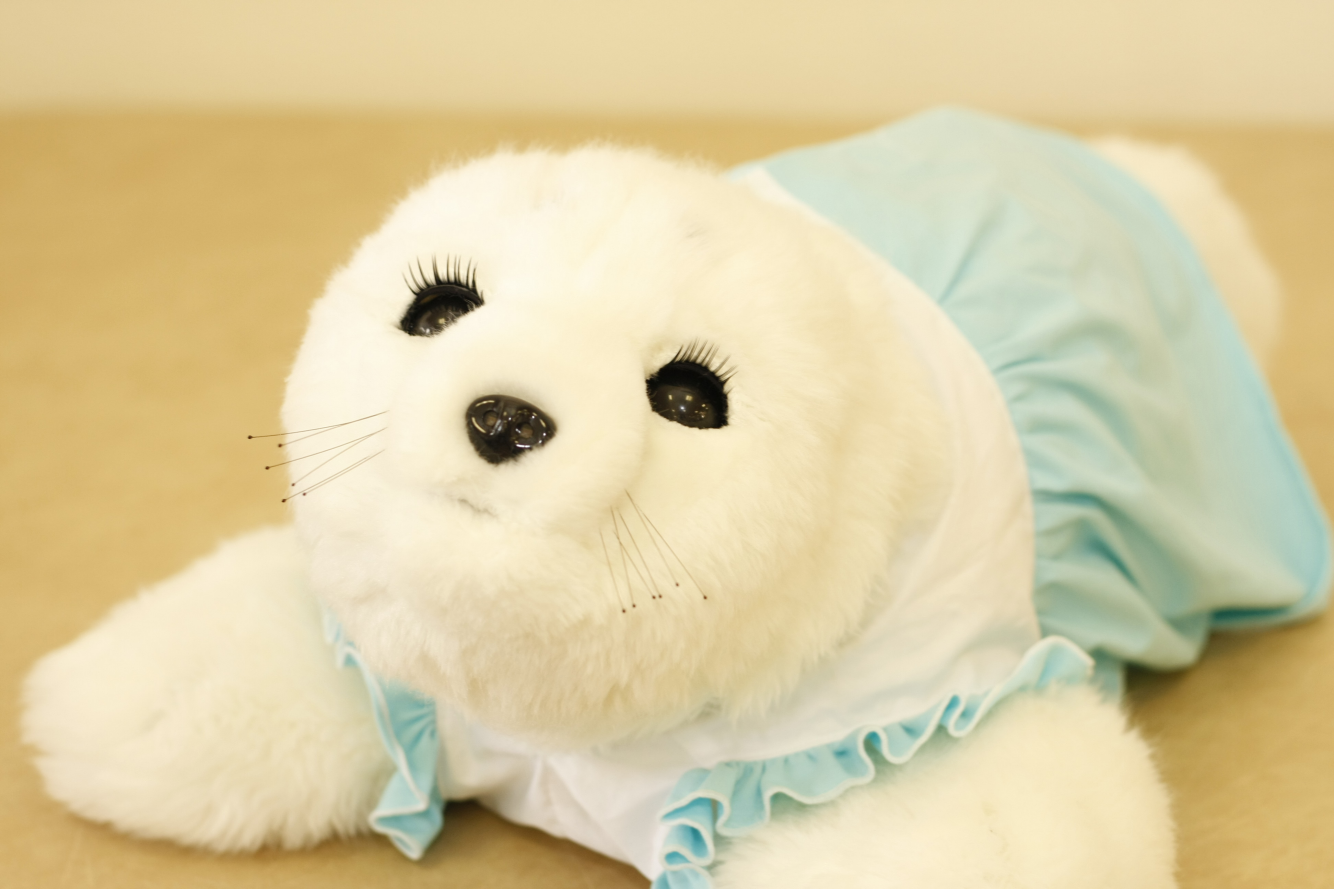
PARO, a social robotic seal.

Amongst the recent literature on the use of socially assistive robots in elderly care, the most widely studied is PARO (Figure 1). PARO is a therapeutic, socially assistive pet-type robot with an appearance of a baby harp seal, and is equipped with different kinds of sensors, including tactile, light, audition, temperature, and posture. Thus, it can respond to different stimulations (eg, striking and holding) given by the users, or recognize the direction of voice from them. It was designed by Shibata et al [18], and has been used with positive and promising results since 2003 in many countries including Japan, Denmark, Canada, Italy, and the United States [19-22]. In 2009, PARO was certified as a type of neurological therapeutic device by the Food and Drug Administration (FDA) in the United States (Registration number: 3009118691) [23]. In 2010, a caregiver’s manual for robot therapy was published to achieve effective therapy [24,25].

Several intervention trials demonstrated promising effects of participating in PARO therapy in increasing motivation, improving mood, reducing stress, and increasing social communication in elderly people [22,26-28]. Positive effects of PARO therapy on mood, social interaction and communication, as well as cortical neuron activity have also been reported in people with dementia [21,29,30]. A recent pilot randomized controlled trial (RCT) reported that for older people with dementia, PARO therapy had a moderate to large positive influence on their quality of life [31]. More recently, the potential therapeutic benefits of PARO for the treatment of neurological diseases have also been published [32]. Although positive effects of PARO therapy in people with dementia or other brain disorders have been reported in various populations, many were observational studies or involved small numbers of subjects. Other interventions, such as animal-assisted therapy, appeared to have beneficial effects on mood, and increased social interaction and communication in people with dementia [33-35]. However, there are many concerns with animal-assisted therapy such as allergies, cleanliness, and the unpredictable nature of live animals.

To date, the potential benefits of PARO therapy in Hong Kong Chinese have not been systematically examined. Given the successful application of PARO therapy, and the encouraging findings of its positive effects on mood and social interaction and communication, it is suggested that PARO therapy is both feasible and acceptable to elderly people with dementia. Therefore, in this study protocol, we describe the design of a RCT aiming to confirm the findings of our pilot data (see Piloting in the Method section), by providing an evaluation of the effectiveness and benefits of the use of PARO in dementia care in Hong Kong.

The primary objective of the present study is to examine whether robot-assisted intervention using PARO in older Chinese adults with mild to moderate dementia improves mood, and stimulates social interaction and communication compared to psychosocial activities by conducting a methodologically rigorous RCT. Secondary objectives are to examine the effects of PARO therapy on cognitive function, depressive symptoms, and caregiver burden compared to those of psychosocial activities.

## Methods

### Study Design

**Figure 2:**
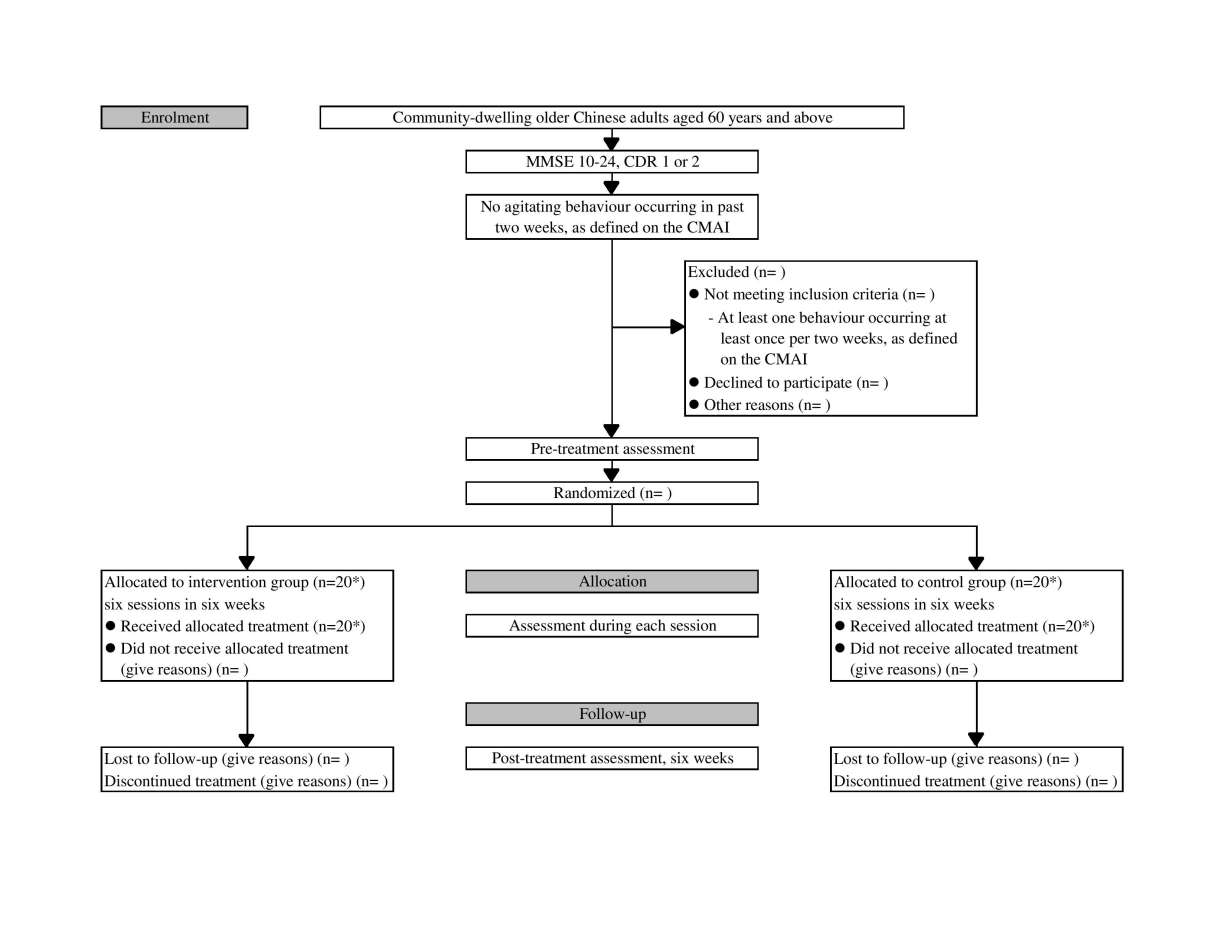
Flow chart of the study design. *Indicates the target number of subjects. CDR: Clinical Dementia Rating, CMAI: Cohen-Mansfield Agitation Inventory, and MMSE: Mini-Mental State Examination.

This is a single-blind RCT comparing PARO therapy and psychosocial activities in the elderly with dementia. Treatment outcomes will be assessed at baseline (pre-), during, and post-treatment. The study will be carried out in Shatin Hospital, a geriatric day hospital located in Shatin, New Territories, Hong Kong. The protocol for this study was registered with the Australian New Zealand Clinical Trials Registry (ACTRN12614000037606) and has been approved by the Clinical Research Ethics Committee of the Chinese University of Hong Kong. The study design is detailed in Figure 2.

### Subjects

#### Overview

The study population will include 40 community-dwelling older Chinese adults with mild to moderate dementia. Recruitment will occur through clinical referrals from community dementia day care centres, geriatric outpatient clinics, nurse-led memory clinics, and day hospitals. Those who are potentially eligible will be invited to a face-to-face screening assessment including an elicitation of a medical history, medications, and hospitalization for eligibility confirmation. To be eligible for the study, subjects must meet the inclusion and exclusion criteria described in the following sections.

#### Inclusion Criteria

Community-dwelling older Chinese adults aged ≥60 years will be screened using the Mini-Mental State Examination (MMSE) [36,37]. Those who score between 10-24 with a diagnosis of dementia will be screened for eligibility to the study. Diagnosis of dementia will be based on the Diagnostic and Statistical Manual of Mental Disorders (DSM-IV). Severity of dementia will be determined using Clinical Dementia Rating (CDR), a widely used clinical staging instrument for dementia [38,39]. Subjects with mild to moderate dementia (CDR 1 or 2) will be further assessed for the presence of agitated behaviour using Cohen-Mansfield Agitation Inventory (CMAI) [40].

####  Exclusion Criteria

Individuals who exhibit any behavioural and psychological symptoms of dementia (at least one item of CMAI scoring ≥2) will be excluded from the study. Subjects will also be excluded if they have severe medical conditions which limit their abilities to complete the course of treatment. Concurrent psychotropic medication will be allowed without restriction, but any change in psychotropic prescriptions over the course of the treatment period will be monitored. In addition, those who are currently participating in other studies, experimental therapies, or blinded treatments will be excluded.

### Informed Consent

For eligible individuals, the study will be introduced to the subjects and their caregivers. Written informed consent will be obtained from every eligible subject agreeing to participate, as well as their caregivers prior to the study. If a subject is unable to give written informed consent, proxy consent will be obtained.

### Baseline Assessment and Randomization

After obtaining written informed consent, a baseline (pre-treatment) assessment will be performed. Subjects will be randomized into either the intervention or the control group. A study coordinator will randomize subjects by means of a computer-generated list of random numbers in blocks of six, stratified by gender. Treatment assignments will be concealed in consecutively-numbered sealed envelopes, which will be opened sequentially upon subject enrollment. As it is a single-blind study, the subjects and the study coordinator will not be blinded to the treatment assignment. However, the study coordinator will not take outcome measurements. All investigators and outcome assessors will be blinded to the treatment assignment.

### Intervention Group

The PARO therapy will take place for 30 minutes once a week over a six week period, and will be delivered in a quiet room that is isolated from the common unit (ward) in the Occupational Therapy Department of Shatin Hospital, with minimal environmental distractions. The number of sessions, the length of each session, and the time frame of this study were decided based on the results of our pilot study (see Piloting of this section) and other PARO intervention studies which reported small or significant changes in mood, social interaction, and communication [15]. Given this, one 30-minute session per week for six consecutive weeks would allow sufficient time for identifying significant changes, and would also reduce the burden of commitment for subjects.

The PARO therapy will be delivered in a structured, small-group approach, in which a group of three to four subjects will be arranged to sit around a table with PARO in the centre. The subjects in each group can be substituted by subjects from other groups to optimize the chance of conducting each session. A facilitator who is familiar with the PARO caregiver’s manual will deliver the PARO therapy. Research team therapists will train the facilitator and monitor the sessions.

The PARO therapy is based upon a standardised framework, and involves activities around the concepts of engaging, social interaction, and communication. There are six stages/themes, including (1) introducing PARO, (2) baby-sitting PARO, (3) grooming PARO, (4) feeding PARO, (5) making over PARO, and (6) wardrobe PARO. During each session, the facilitator will show PARO to each subject and demonstrate how PARO responds. Subjects will be encouraged to touch and hold PARO, describe the features and appearance of PARO, and help take care of PARO. The session will proceed for at least 30 minutes. With one session per week, there will be at least a total of 180 minutes of the treatment in the six week period.

### Control Group

Subjects assigned to the control group will be invited to practise a variety of psychosocial activities including a range of table games (eg, Chinese checkers, Jenga, board games etc). The activities will be held on exactly the same schedule as the intervention group (one 30-minute session per week for six weeks). All activities will be carried out in groups of three to four subjects, and will be facilitated by the same facilitator of the intervention group. The sessions will proceed for at least 30 minutes. With one session per week, there will be at least a total of 180 minutes of the treatment in the six week period.

### Outcome Measures

Measurement of outcomes will take place at pre-, during, and post-treatment. Mood, social interaction, and communication (ie, facial expressions and reactions towards each treatment) will be the primary outcome measures. Secondary outcome measures will include cognitive function and depressive symptoms, as well as caregiver burden. Pre-treatment and post-treatment assessments will be administered by a trained research assistant who will remain blinded to group allocation. Outcome measures administered at each time point are described in Table 1. Attendance at each session will be recorded for all subjects in the study.

**Table 1:** Schedule of assessments.

Assessments	Screening	Pre-treatment	Treatment session number						Post-treatment
			1	2	3	4	5	6	
MMSE^a^	√ ^g^								√
CDR^b^	√								
CMAI^c^	√								
Demographics, lifestyle, and social characteristics		√							
Simplified face scale			√	√	√	√	√	√	
Observation table^d^			√	√	√	√	√	√	
CSDD^e^		√							√
ZBI^f^		√							√
Subjective impression questionnaire			√	√	√	√	√	√	
Qualitative comments from caregivers, facilitator, and therapists									√

^a^Mini-Mental State Examination (MMSE)

^b^Clinical Dementia Rating (CDR)

^c^Cohen-Mansfield Agitation Inventory (CMAI)

^d^Facial expressions and reactions towards each treatment

^e^Cornell Scale for Depression in Dementia (CSDD)

^f^Zarit burden interview (ZBI)

^g^The √ indicates at which point of the study the respective assessments will take place.

### Demographics, Lifestyle, and Social Characteristics

Demographic information such as age, gender, marital status, educational background, living status, lifestyle factors (eg, smoking and alcohol intake), social supporting network (eg, measures of participation in day care centre, cognitive/memory training, community activities, and supporting groups), attributes of the owners of pet (ie, animal preference, ever kept pets, and plan to keep pets) [41], and child care experiences will be extracted from pre-treatment questionnaires.

### Primary Outcomes

#### Simplified Face Scale

A simplified face scale will be used to assess mood state before and after each session. This very brief, pictorial scale of mood uses a sequence of 7 faces and does not require reading literacy [27]. The original face scale contains 20 drawings of a single face arranged in serial order by rows, with each face depicting a slightly different mood state [42]. However, sometimes the subjects were confused by the original face scale because it contained too many similar images. Thus, the scale was simplified by using only 7 images from the original set. The 7 faces range from very happy at the left to very sad at the right.

#### Observation Table

In order to objectively examine changes in subjects’ social interaction and communication (ie, facial expressions, and reactions towards PARO or the psychosocial activities), treatment sessions will be videotaped and charted on an every-minute basis with an observation table by reviewing the videotaped clips after each treatment session. The observation table is a modified version of the one originally developed by Wada et al [24]. Responses will be classified into several categories, including expression, gaze, and interactions with PARO or the psychosocial activities, other subjects, facilitator, and therapists. The frequency of each response will be added, and depending on the duration of each session, the highest possible item-score is 30. The amount of time spent on verbal encouragement (prompting) offered will also be recorded.

To assess the inter-rater and intra-rater reliabilities of the modified observation table, a convenience sample of 11 community-dwelling older Chinese adults with mild cognitive impairment or mild to moderate dementia with a mean age of 80.5 (range 73-88 years, SD 4.9), of which 82% (9/11) were female, and with a mean MMSE score of 16.5 (range 9-24, SD 5.9) were recruited to receive a single 30-minute PARO therapy. The therapy sessions were videotaped and observed by two raters (one occupational therapist and one medical researcher) who independently marked the subjects’ facial expressions and reactions towards PARO on the modified observation table on an every-minute basis (with standardized rating criteria), on two different occasions with an interval of two to four weeks. Intraclass correlation coefficient (ICC) was used to measure the reliability of the ratings. The ICC of the inter-rater reliability was.95-1.00 and the ICC of the intra-rater reliability was.87-1.00 [43].

### Secondary Outcomes

#### Mini-Mental State Examination (MMSE)

The Mini-Mental State Examination (MMSE) is a validated scale to assess cognitive performance in both research and clinical settings [36]. It contains 20 items and scores range from 0-30, with a higher score denoting better cognitive function. The Chinese version of the MMSE has good psychometric properties, with satisfactory internal consistency (Cronbach’s alpha=.86) and test-retest reliability (alpha=.78), and good inter-rater reliability (ICC=.99) [37].

#### Cornell Scale for Depression in Dementia (CSDD)

The Cornell Scale for Depression in Dementia (CSDD) is a validated scale used to assess the signs and symptoms of major depression in patients with dementia [44]. The information is elicited through two semi-structured interviews, one with the patient and one with the caregiver. The scale is divided into the following five sub-scales (1) mood-related signs, (2) behavioural disturbance, (3) physical signs, (4) cyclic functions, and (5) ideational disturbance. There are 19 items, of which each can be given a score ranging from 0 (absent) to 2 (severe). The total score ranges from 0-38; a higher score denotes greater levels of depression. The Chinese version of the CSDD demonstrated satisfactory internal consistency (Cronbach’s alpha=.84) and inter-rater reliability (kappa=.43-.89) [45,46].

#### Zarit Burden Inventory (ZBI)

The Zarit Burden Inventory (ZBI) is a validated scale to assess caregiver burden [47]. It contains 22 items, each of which can be given a score ranging from 0 (never) to 4 (nearly always). The total score ranges from 0-88; a higher score denotes greater perceived caregiver burden. The Chinese version of the ZBI showed a good internal consistency reliability (ICC=.99, split half correlation coefficient=.81) [48].

### Other Assessments

#### Subjective Impression Questionnaire

Subjective impression of PARO will be assessed at the end of each session of the PARO therapy by adopting four items from studies conducted by Shibata et al [29,49], with two of them reflecting subjects’ preference towards PARO and the other two for understanding their own feelings when interacting with PARO and their readiness to receive the PARO therapy. The four questions are (1) Is PARO cute/ugly?, (2) Do you like/dislike PARO?, (3) Is playing with PARO fun or boring?, and (4) Do you want to play with PARO again? For the group assigned to the psychosocial activities, four similar questions will be used (1) Is the activity interesting/not interesting?, (2) Do you like/dislike the activity, (3) Is the activity fun or boring?, and (4) Do you want to join the activity again?

#### Qualitative Comments

Qualitative comments from the caregivers, the facilitator, and the therapists will also be obtained from semi-structured qualitative interviews. Interviews will be conducted within one month of completing the treatment program. For the caregivers, the interviews will explore their perceptions of the impact of dementia on the subjects’ daily lives, experiences of the interventions that were designed to improve mood and encourage social interaction and communication since diagnosis, and feedbacks after the treatments. For the facilitator and the therapists, the interviews will elicit information about the subjects’ reactions towards each treatment. Furthermore, the challenges of subject recruitment, treatment implementation, as well as the factors associated with interest in engagement and adherence of the treatments will also be obtained.

### Piloting

**Figure 3:**
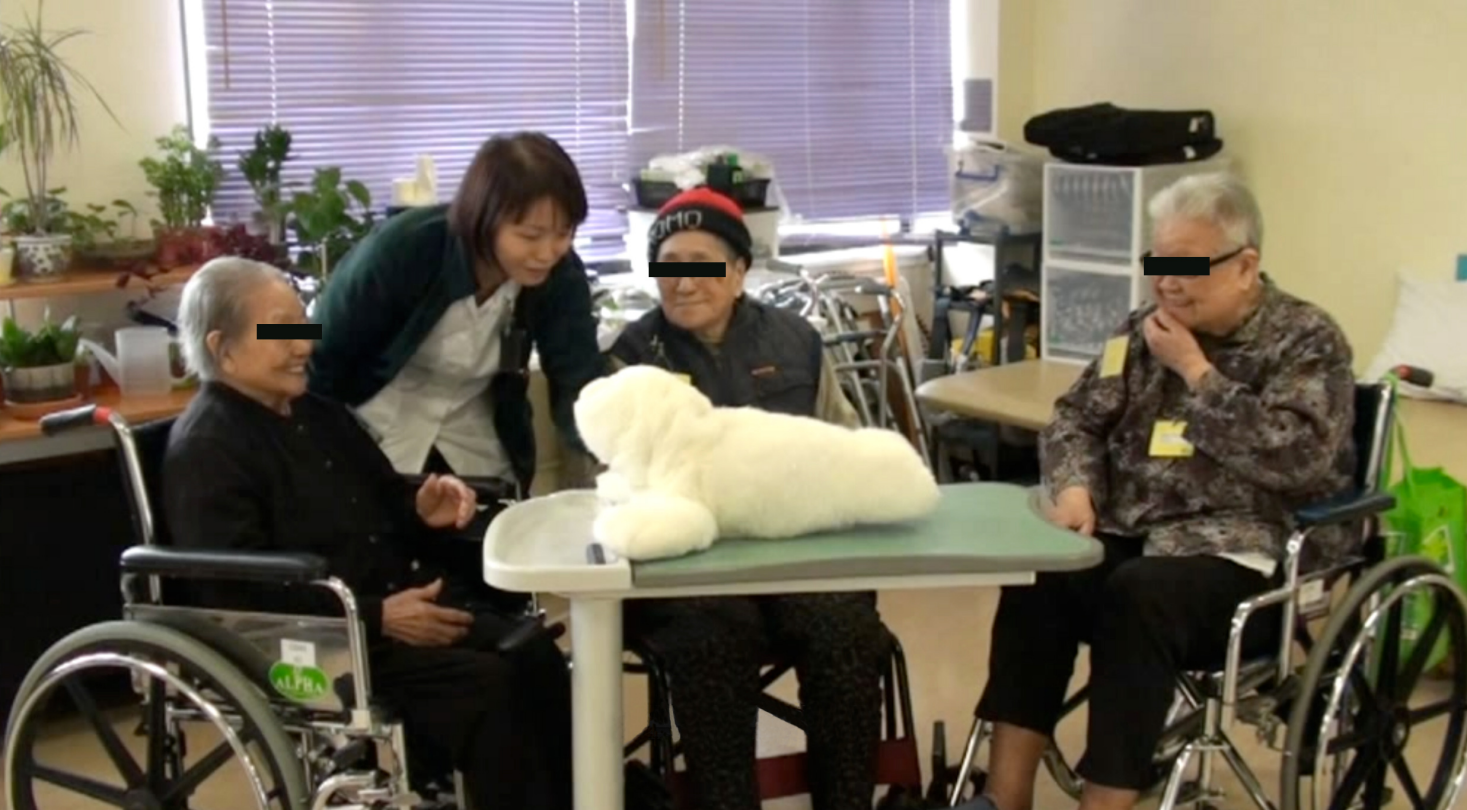
PARO therapy.

Prior to the study, a pilot study was performed to explore the feasibility and potential benefits of PARO therapy in older adults with dementia. Using a pre-post single group design, community-dwelling older Chinese adults with mild cognitive impairment or mild to moderate dementia were recruited to receive six sessions of the PARO therapy (one 30-minute session per week for six consecutive weeks) in Shatin Hospital. The PARO therapy was delivered in a structured, small-group approach, in which a group of 3-4 subjects were arranged to sit around a table with PARO in the centre. An occupational therapist familiar with the PARO caregiver’s manual delivered the PARO therapy (Figure 3). The mean age of the 7 subjects that completed the PARO therapy was 78.6 (range 72-87, SD 5.3), of which 43% (3/7) were female, and with a mean MMSE score of 19.3 (range 16-22, SD 2.8). Using the Wilcoxon signed ranks test, there was a significant improvement in mood, as evaluated by the simplified face scale, following the PARO therapy (*P*=.02). Social interaction and communication was evaluated by video analysis of facial expressions and reactions towards PARO using a time sampling method where the 30 minutes of video were divided into 30 units, and each one minute unit was checked for the occurrence of each facial expression and reactions towards PARO. The analysis showed that the frequency of neutral expressions during the six 30-minute sessions was high (mean observed frequency of the six sessions was 27 minutes out of 30 minutes), followed by smile (13 minutes out of 30 minutes), and laugh (9 minutes out of 30 minutes). Furthermore, all subjects gently stroked or held PARO during the interaction, and talked directly with PARO in a dyadic relation as if it was a real living pet. In addition, there was a positive trend in depressive symptoms, as evaluated by CSDD (*P*=.03) and a falling trend in caregiver burden, as evaluated by ZBI (*P*=.02) immediately following the PARO therapy. All subjects completed the six-session PARO therapy, with the attendance rate of 100%. Thus, our findings provide important preliminary support for the use of social robot in engaging older patients with dementia in a day care setting. The questionnaires and assessment protocols have been pilot-tested.

### Sample Size Calculation

Sample size calculations were based on the effects on mood in the pilot study: a sample size of 15 subjects per group will allow us to detect differences among the mean values of face scale scores (0.63, SD 0.74) between pre- and post-interventions using one sample *t* test (alpha=.05 and power=.9). Given that the attrition rate of 20% was observed in a previous PARO study of people with dementia [30], we will estimate a more conservative attrition rate of 25%, and thus we will recruit a total of 40 subjects.

### Statistical Analysis

Double data entry, consistency check, and data cleaning will be performed prior to data analysis. An intention-to-treat (ITT) analysis will be carried out, in that all available data will be included, without considering the subjects’ compliance to the allocated treatment. Mean and standard deviation will be used for continuous variables while frequency and percentage will be used to describe the distribution of ordinal and categorical variables. Unpaired tests will be used for the primary analysis. Differences between the intervention and control groups in relation to outcome measures will be compared using an analysis of variance (ANOVA) or independent *t* tests. In order to increase precision in estimating the effect of interest, the analysis of covariance (ANCOVA) will be used to take into account the possible confounding effects of the covariates, such as age, education, etc. A mixed-model, repeated-measures ANCOVA will be used to compare the reactions towards each treatment captured by the observation table in both groups. The effect size will be computed to show the magnitude and direction of the effect of the intervention group relative to the control condition for each outcome variable. Data analyses will be conducted by SPSS Statistics software. A *P*<.05 will be taken as the level of statistical significance. The 95% confidence interval around the differences will be calculated.

## Results

Recruitment to the pilot study began in 2014 and the last subject is expected to complete the post-treatment assessment in 2015.

## Discussion

### Significance of the Study

The proposed study is, to our knowledge, the first RCT of the use of PARO in improving mood, and stimulating social interaction and communication compared to psychosocial activities in older Chinese adults with mild to moderate dementia in a day care setting in Hong Kong. The results could have particular importance given the rise in the prevalence of dementia in our society.

### Study Strengths and Limitations

The proposed study has several notable methodological strengths. The use of videotaped observations will allow us to capture subjects’ facial expressions and reactions towards each treatment during the treatment sessions, of which standard questionnaires or proxy interviews may miss. The modified observation table assesses the degree to which people with dementia will respond with the treatment by videotaped observation, which has the advantage in that ratings were specifically developed in the context of social interaction and communication between elderly patients with dementia and PARO or the psychosocial activities and other subjects, the facilitator, and therapists. Inter-rater and intra-rater reliabilities of the table have been developed. Another strength of the proposal is the measurement of various variables including caregiver burden, and qualitative components from the caregivers, facilitator, and therapists, which could reflect mood and social behaviours in another perspective.

There are several limitations in the protocol. Although behavioural and psychological symptoms of dementia occur frequently in people with dementia, we will confine our study population to mild to moderate dementia, and will exclude those with behavioural and psychological symptoms of dementia, limiting the generalizability of the results to a wider population. It has been suggested that interventions for those with behavioural and psychological symptoms of dementia should tailor the person’s specific needs and capabilities [50]. As such, an individual-based setting may be more appropriate to those with behavioural and psychological symptoms of dementia, while a group-based setting is preferred to examine the use of PARO in stimulating social interaction and communication. Moreover, subjects will be recruited through clinical referrals from community day care centres, clinics, and day hospitals; they may not represent all older adults with dementia in the community. In addition, the simplified face scale, CSDD, ZBI, and the subject impression questionnaire are self-reported. Misreporting and non-reporting may occur.

### Conclusions

This proposed study will provide an evaluation of the effectiveness and benefits of the use of PARO in older Chinese adults with mild to moderate dementia in a day care setting. Results of this study would showcase a novel activity to improve mood, and stimulate social interaction and communication in community care of older people with dementia, as well as provide an evidence base for the use of such social robots. Further research is warranted to examine the use of PARO in managing behavioural and psychological symptoms of dementia using individualized approaches.

## Abbreviations

ANCOVA: analysis of covariance
ANOVA: analysis of variance
CDR: clinical dementia rating
CMAI: Cohen-Mansfield Agitation Inventory
CSDD: Cornell Scale for Depression in Dementia
DSM-IV: diagnostic and statistical manual of mental disorders
ICC: Intraclass correlation coefficient
ITT: intention-to-treat
MMSE: Mini-Mental State Examination
RCT: randomized controlled trial
ZBI: Zarit Burden Inventory
